# Connectome-based mapping of gray matter abnormalities in hepatic encephalopathy

**DOI:** 10.3389/fmed.2026.1886303

**Published:** 2026-07-10

**Authors:** Li Chen, Lei Xia, Yaling Chen, Chengkun Hong, Minghui Mao, Xiaoyang Wang, Lili Zhou

**Affiliations:** 1 Department of Hepatobiliary Disease, Fuzong Clinical Medical College of Fujian Medical University, Fuzhou, Fujian, China; 2 Department of Hepatobiliary Disease, The Third Affiliated People’s Hospital of Fujian University of Traditional Chinese Medicine, Fuzhou, Fujian, China; 3 Department of Radiology, Fuzong Clinical Medical College of Fujian Medical University, Fuzhou, Fujian, China; 4 Department of Diagnostic Radiology, Fuzong Teaching Hospital of Fujian University of Traditional Chinese Medicine (900th Hospital), Fuzhou, Fujian, China; 5 Department of Hepatobiliary Disease, 900th Hospital of PLA Joint Logistic Support Force, Fuzhou, Fujian, China

**Keywords:** brain connectome, disease epicenter, gray matter abnormalities, hepatic encephalopathy, network modeling, structural connectivity

## Abstract

**Objective:**

Hepatic encephalopathy (HE) is linked to widespread gray matter abnormalities, but it remains unclear whether these changes follow the organizing principles of large-scale brain networks. This study examined the spatial distribution of gray matter abnormalities in HE and their relationships with brain network hubs, neighborhood connectivity, and disease epicenters.

**Methods:**

In this cross-sectional study, 45 patients with HE and 45 healthy controls underwent high-resolution T1-weighted MRI. Cortical thickness was extracted from 308 cortical regions using the Desikan–Killiany atlas, and volumes were measured from 14 subcortical structures. Group differences were analyzed controlling for age, sex, and total intracranial volume. Connectome-based analyses were based on normative functional and structural connectomes from the Human Connectome Project and assessed hub-related vulnerability, network-neighborhood effects, disease epicenters, and individual-level network patterns.

**Results:**

Patients with HE showed widespread gray matter abnormalities in the prefrontal, motor, temporal, and limbic cortices, as well as basal ganglia and thalamus, without significant alignment with normative functional or structural hubs. Structural neighborhood abnormalities were positively correlated with cortical changes (*r* = 0.58, P_*spin*_ = 0.004), whereas functional neighborhoods were not. Functional-connectome epicenters were concentrated in the left inferior frontal gyrus, orbitofrontal cortex, and striatum, while structural-connectome epicenters centered on the bilateral superior frontal gyri and left inferior frontal gyrus. Individual analyses revealed heterogeneous epicenter patterns, with prefrontal-related regions repeatedly implicated.

**Conclusion:**

These findings suggest that gray matter abnormalities in HE are non-randomly organized, constrained by structural connectivity, and associated with prefrontal-centered disease epicenter networks, providing connectome-based insights into gray matter abnormalities in HE.

## 1 Introduction

Hepatic encephalopathy (HE) is a major neuropsychiatric complication of liver cirrhosis, manifesting as subtle cognitive deficits, overt consciousness impairment, or coma. HE is an important clinical marker of hepatic decompensation and is strongly associated with reduced quality of life, increased hospital readmission, and higher mortality risk (1). Neuroimaging studies indicate that HE is not a focal brain injury but involves widespread structural and functional remodeling, affecting cortical thickness, gray matter volume, edema-related signals, and various functional network metrics (2, 3). However, most neuroimaging studies of HE have focused on individual regions, networks, or modalities, leaving it unclear whether gray matter abnormalities follow principles of whole-brain spatial organization. Therefore, elucidating the neurobiological basis of HE from the perspectives of whole-brain structural organization and network architecture is important for understanding its pathological mechanisms and may help generate hypotheses for future longitudinal studies of clinically validated imaging markers.

Brain connectome research provides an important theoretical framework for explaining the spatial distribution of disease-related gray matter abnormalities. In network neuroscience, the brain is conceptualized as a complex communication system in which structural connectivity (SC) and functional connectivity (FC) support interregional information integration. Consequently, disease-related injury is often non-random and may follow specific spatial patterns along pre-existing connectivity architectures (4, 5). Within this framework, highly connected hub nodes may be more vulnerable to pathology due to higher information integration and metabolic demand (6). Furthermore, abnormality severity in a region may correlate with the mean abnormality burden of its connectivity-defined neighborhood, indicating a network-neighborhood constraint (7). Moreover, disease epicenter localization identifies key regions whose connectivity profiles explain the whole-brain distribution of abnormalities, providing insight into network anchors of disease-related alterations. Recently, this framework has been validated across various brain disorders (8–10). However, despite widespread neuroimaging abnormalities in HE, studies systematically evaluating hub vulnerability, network neighborhood constraints, and disease epicenter distribution based on normative connectomes remain limited.

**FIGURE 1:**
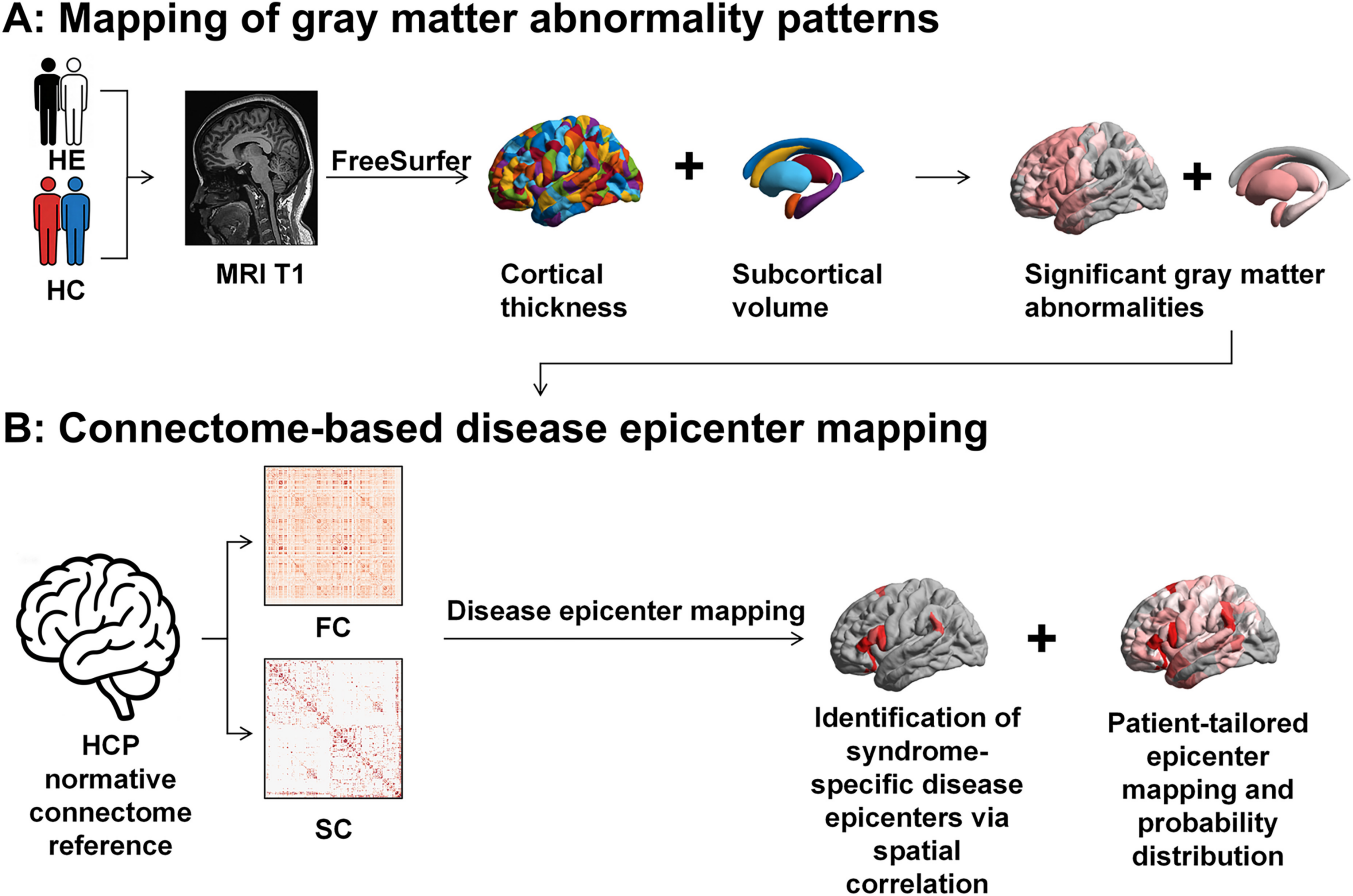
Overall analytical framework of the study. **(A)** Group-level analysis pipeline. T1-weighted MRI data from patients with HE and HC were processed with FreeSurfer to obtain cortical thickness and subcortical volume measures, and group-level gray matter abnormalities were identified by comparing HE with HC. **(B)** Connectome-based modeling pipeline. Group-level abnormality maps were combined with normative FC and SC from the HCP to test hub vulnerability, network-neighborhood effects, disease epicenters, and patient-tailored epicenter patterns. HE, hepatic encephalopathy; HC, healthy controls; FC, functional connectivity; SC, structural connectivity; HCP, Human Connectome Project.

Against this background, we used T1-weighted structural MRI to delineate group-level gray matter abnormality maps in patients with HE. We further integrated normative FC and SC networks from the Human Connectome Project (HCP) (11) to evaluate the relationships between HE-related gray matter abnormalities and hub distribution, neighborhood constraints, disease epicenters, and individual-level epicenter patterns. The analytical framework is illustrated in Figure 1. Specifically, we examined whether gray matter abnormalities in HE follow non-random whole-brain spatial patterns, whether they are primarily constrained by FC or SC, whether key disease epicenters explain the whole-brain abnormality distribution, and whether group-level network features are reproducible at the individual level. These analyses aim to reveal the network basis of HE-related gray matter abnormalities from a connectome perspective and provide new insights into the neuroimaging heterogeneity of HE.

## 2 Materials and methods

### 2.1 Study participants

This cross-sectional study included 45 patients with HE and 45 healthy controls (HC). Patients were classified as HE if they had either a documented episode of overt HE within 1 month before MRI or evidence of HE at enrollment. HE status at enrollment was categorized as covert HE or overt HE. Covert HE included minimal HE and West Haven grade 1 HE; minimal HE was defined as West Haven grade 0 with a Psychometric Hepatic Encephalopathy Score (PHES) ≤−4. Overt HE at enrollment was defined as West Haven grade 2. Patients with West Haven grade 3–4 were not included because they were unable to complete MRI and neuropsychological assessment reliably. The main exclusion criteria were alcohol-related cirrhosis or heavy alcohol intake within the past 6 months, current psychoactive substance use disorder, age younger than 18 years or older than 75 years, contraindications to MRI, concomitant hepatic malignancy, rapidly progressive liver failure, visible structural brain lesions on conventional MRI, poor MRI image quality, or incomplete key demographic information. Participants in the HC group were recruited from the Health Examination Center of the same hospital and had no history of chronic liver disease, neurological disease, psychiatric disorder, major systemic disease, or contraindications to MRI. Neuropsychological performance was summarized using the Mini-Mental State Examination (MMSE), Montreal Cognitive Assessment (MoCA), and PHES. The study was approved by the Biomedical Ethics Committee of the 900th Hospital of PLA Joint Logistic Support Force (Approval No. 2026-118), and all participants provided written informed consent.

### 2.2 Neuroimaging data acquisition and preprocessing

High-resolution T1-weighted images were acquired on a United Imaging uMR 770 3.0-T scanner with a 32-channel phased-array head coil. The main acquisition used a 1.0-mm isotropic three-dimensional T1-weighted structural sequence; full scanner parameters are listed in the Supplementary material. Raw images were processed with FreeSurfer v8.1.0 (12). The processing stream included skull stripping, tissue segmentation, cortical surface reconstruction, and spherical registration. All reconstructions underwent visual quality control of cortical surfaces and segmentation outputs, and cases with segmentation errors or surface inaccuracies were manually corrected and reprocessed.

### 2.3 Extraction of morphological metrics and group-level comparison

Cortical thickness was extracted from 308 cortical regions, and volumes were extracted from 14 subcortical gray matter structures based on FreeSurfer reconstruction and the refined Desikan-Killiany 308-region (DK308) atlas (13, 14). Group-level gray matter differences between HE and HC groups were tested with linear models in SurfStat (15), with group as the main effect and age, sex, and total intracranial volume as covariates. For each region, the *t*-value and uncorrected *P*-value from the between-group comparison were retained. Negative *t*-values indicated lower cortical thickness or subcortical volume in patients with HE than in controls, whereas positive *t*-values indicated higher cortical thickness or subcortical volume in patients with HE than in controls. Multiple comparisons were controlled using the false discovery rate (FDR), with P_*FDR*_ < 0.05 considered significant. To assess the robustness of our findings to the choice of cortical parcellation, part of the analysis was also repeated using the Schaefer400 parcellation (16) and quantitative consistency metrics were reported in the Supplementary material.

### 2.4 Spatial permutation testing

Spatial permutation testing was used because neighboring brain regions are not statistically independent and standard spatial correlations may overestimate significance (17). Cortical map associations were assessed with spin permutation testing, whereas subcortical associations were assessed with random label permutation because spherical rotation is not suitable for subcortical structures (8). Unless otherwise stated, 10,000 permutations were used to generate null distributions for group-level spatial analyses. Implementation details are provided in the Supplementary material.

### 2.5 Generation of HCP normative networks

To test whether HE-related gray matter abnormalities follow normative connectome organization, reference connectivity networks were generated from unrelated healthy adults in the HCP (11). Following the connectome framework used in previous network-based morphometric modeling studies, the HCP normative sample comprised 207 unrelated healthy adults (83 males and 124 females), with a mean age of 28.73 ± 3.73 years and an age range of 22–36 years. The HCP dataset included high-resolution T1-weighted MRI, resting-state functional MRI, and diffusion MRI acquired on a Siemens Skyra 3.0-T scanner. HCP data were processed with the standard project pipelines (18), and regional FC and SC matrices were reconstructed using the same parcellation scheme as in the HE analysis. The HCP connectomes were used as healthy normative reference networks to assess whether gray matter abnormalities observed in patients with HE were spatially aligned with canonical connectome architecture. Detailed HCP preprocessing steps are provided in the Supplementary material.

### 2.6 Generation of FC and SC matrices

The normative FC matrix was generated from pairwise Pearson correlations among regional resting-state functional MRI time series. FC was calculated among cortical regions and between cortical and subcortical regions. Negative correlations were set to zero to focus on positive interregional coupling and to avoid ambiguity in the interpretation of negative edge weights in weighted-degree, network-neighborhood, and disease epicenter analyses. Individual FC matrices were Fisher z-transformed before averaging across HCP participants to construct the group-level normative FC matrix.

Normative SC was reconstructed from diffusion MRI using MRtrix3 (19). Streamlines were generated using anatomically constrained tractography and weighted using spherical-deconvolution informed filtering of tractograms 2 (SIFT2) (20). Reconstructed streamlines were mapped to the 308 cortical and 14 subcortical regions. For each pair of regions, SC strength was first calculated as the sum of SIFT2 weights of all streamlines connecting the two regions. A distance-dependent thresholding strategy was used to construct the group-level SC matrix while preserving individual edge-length distributions (21). The resulting SC weights were log-transformed to reduce connectivity strength variance and the influence of extremely strong edges. Therefore, the SC weights used in the hub-related, network-neighborhood, and disease epicenter analyses represented log-transformed SIFT2-weighted SC values rather than raw streamline counts. No additional arbitrary proportional thresholding was applied in the subsequent hub-related, network-neighborhood, or disease epicenter analyses. Tractography parameters and additional matrix construction details are provided in the Supplementary material.

### 2.7 Hub-related analysis

Hub-related vulnerability was tested by spatially correlating the group-level HE abnormality map with weighted-degree maps derived from normative FC and SC. Degree was defined as the sum of connection weights between a given region and all other regions, with higher values indicating stronger hub-like topology. Cortical and subcortical systems were analyzed separately, and Spearman correlations were used for all hub analyses.

### 2.8 Network neighborhood constraint analysis

Network-neighborhood analysis examined whether abnormality in a region was related to the abnormality burden of its connected neighbors. Following previous connectome-based morphometric modeling work (22), we calculated a degree-normalized connection-weighted neighbor abnormality for each brain region (i) as follows:


$$A_{i}=\frac{1}{N_{i}}\,\sum_{j=1}^{N_{i}}a_{j}\,W_{\mathrm{ij}},j\neq i$$

Here, A_i_ denotes the degree-normalized connection-weighted neighbor abnormality of region (i); a_j_ denotes the group-level gray matter abnormality of connected neighbor (j); W_*ij*_ denotes the FC or SC weight between regions i and j; and N_i_ denotes the number of neighbors connected to region i. Subsequently, at both cortical and subcortical levels, Spearman correlations were computed between each region’s abnormality burden and its FC- and SC-weighted mean neighbor burden. A standard weight-normalized sensitivity analysis is described in the Supplementary material. These analyses assessed whether HE-related gray matter abnormalities exhibit network constraint features. Significance was tested using spin permutation for cortical regions and random label permutation for subcortical regions.

### 2.9 Disease epicenter localization analysis

Disease epicenter localization was performed to identify regions whose normative connectivity profiles best explained the spatial distribution of the whole-brain HE gray matter abnormality map. For each cortical and subcortical region, seed connectivity profiles were extracted from the normative functional and structural matrices and spatially correlated with the group-level gray matter abnormality map. Regions with top-ranked and statistically significant correlations were operationally defined as candidate disease epicenters, with significance assessed using spin permutation for cortical regions and random label permutation for subcortical regions. Disease epicenter analyses used the resulting weighted connectivity matrices without applying an additional arbitrary proportional threshold to reduce threshold-dependent bias. We also correlated epicenter maps with degree maps to test whether epicenters were topologically related to hubs.

### 2.10 Individual-level network model analysis

Individual-level network model analysis was used to assess whether group-level network features were also detectable within patients. Each patient’s cortical thickness and subcortical volume values were converted to z scores relative to the HC group to create individualized abnormality maps. Individual hub analyses correlated each patient map with normative functional and structural degree maps. Individual epicenter analyses correlated each patient map with normative seed connectivity profiles for all regions; regions reaching permutation-based significance were considered candidate individual disease epicenters. The hit rate of each region was defined as the percentage of patients in whom that region was identified as a significant epicenter. Additional individual-level permutation settings are provided in the Supplementary material.

## 3 Results

### 3.1 Baseline characteristics of the study participants

The final sample included 45 patients with HE and 45 individuals in the HC group. Detailed demographic and clinical characteristics are summarized in Table 1. The groups did not differ significantly in age (*t* = 1.825, *P* = 0.071), sex distribution (χ^2^ = 0.814, *P* = 0.367), or total intracranial volume (*t* = 0.833, *P* = 0.407), indicating reasonable baseline comparability. Compared with the HC group, patients with HE showed significantly lower MMSE, MoCA, and PHES scores, indicating impaired global cognitive and psychometric performance in the HE group.

**TABLE 1:** Demographic and clinical characteristics of the study participants.

Characteristic	HE group (*n* = 45)	HC group (*n* = 45)	*P*-value
Age (y)*	58.58 ± 7.75	55.53 ± 8.08	0.071
Sex, *n* (%)			0.367
Male	33 (73.3)	28 (62.2)	
Female	12 (26.7)	17 (37.8)	
Education (y)*	8.07 ± 2.61	8.22 ± 2.85	0.788
Total intracranial volume (mm^3^)*	1454791.02 ± 130051.92	1431439.13 ± 135817.35	0.407
HE subtype, *n* (%)		/	/
Covert HE	10 (22.2)	/	
Overt HE	35 (77.8)	/	
Etiology, *n* (%)		/	/
Viral hepatitis	34 (75.6)	/	
Autoimmune	10 (22.2)	/	
Mixed (viral hepatitis + autoimmune)	1 (2.2)	/	
Lactulose use at MRI, *n* (%)	35 (77.8)	/	/
MMSE^#^	27 (26, 28)	30 (30)	<0.001
MoCA^#^	26 (24, 28)	30 (29, 30)	<0.001
PHES^#^	−5 (−6, −3)	0 (0, 0)	<0.001
Ammonia (μmol/L)*	95.11 ± 39.35	/	/
Child-Pugh scores#	9 (7, 10)	/	/
Model for end-stage liver disease scores*	13.16 ± 4.29	/	/
Albumin (g/L)*	31.31 ± 5.01	/	/
Total bilirubin (μmol/L)^#^	36.10 (21.10, 62.90)	/	/
International normalized ratio#	1.27 (1.22, 1.41)	/	/
Sodium (mmol/L)^#^	141.0 (138.0, 142.4)	/	/
Creatinine (μmol/L)^#^	67.0 (56.5, 85.7)	/	/

*Data are means ± standard deviations.

^#^Data are medians, with interquartile ranges in parentheses. *P*-values were calculated for variables available in both groups. Independent-sample t tests were used for normally distributed continuous variables, Mann–Whitney U tests were used for non-normally distributed continuous variables or ordinal variables, and chi-square tests with Yates correction were used for sex distribution. HE, hepatic encephalopathy; HC, healthy controls; MMSE, Mini-Mental State Examination; MoCA, Montreal Cognitive Assessment; PHES, Psychometric Hepatic Encephalopathy Score.

### 3.2 Group-level gray matter abnormality map in HE

**FIGURE 2:**
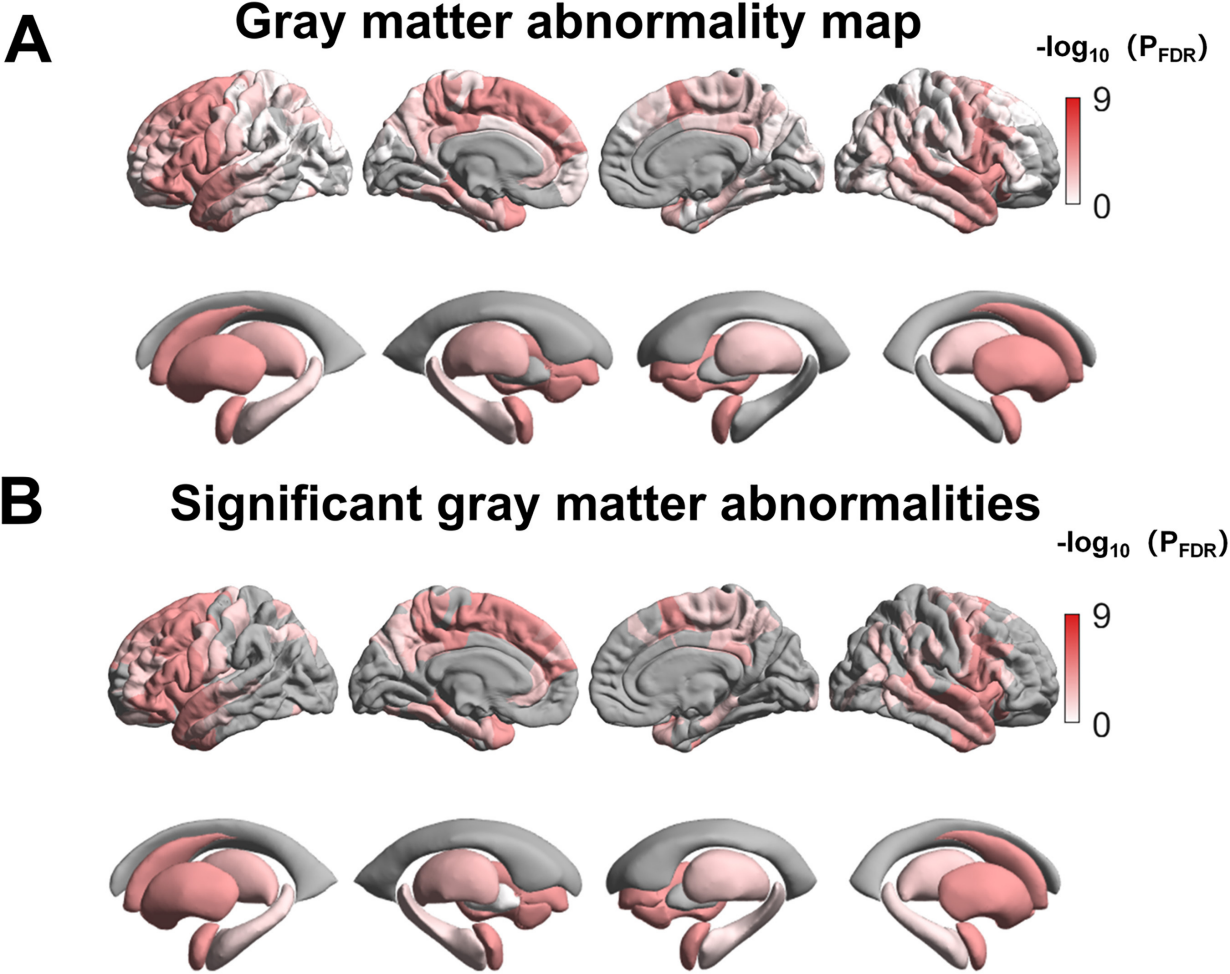
HE is associated with widespread cortical and subcortical gray matter abnormalities. **(A)** Unthresholded group-level gray matter abnormality map showing regional cortical thickness and subcortical volume differences between HE and HC. Cortical maps are displayed on the brain surface, and subcortical maps are shown separately. **(B)** Regions surviving FDR correction. Color bars indicate -log10(P_*FDR*_), with higher values indicating stronger statistical evidence for group differences. HE, hepatic encephalopathy; HC, healthy controls; FDR, false discovery rate.

T1-weighted MRI revealed a broad pattern of gray matter abnormalities in HE. Across 322 cortical and subcortical regions, 167 regions survived FDR correction (Supplementary Table 1), and 154 of these showed lower cortical thickness or subcortical volume in patients than in controls. The most prominent regions with lower cortical thickness or subcortical volume involved the left inferior, middle, and superior frontal gyri, bilateral precentral gyri, bilateral superior temporal regions, posterior cingulate cortex, and insula (Figure 2A). Subcortical abnormalities were mainly located in the bilateral amygdala, caudate nucleus, putamen, nucleus accumbens, thalamus, and left hippocampus (Figure 2B). Overall, gray matter abnormalities observed in patients with HE extended across prefrontal, motor, temporal, limbic, basal ganglia, and thalamic systems. Repeating the group analysis with the Schaefer400 parcellation produced a similar distributed pattern with comparable effect directionality (Supplementary Figure 1 and Supplementary Table 4).

### 3.3 Spatial relationship between HE gray matter abnormalities and hub distribution in normative brain networks

**FIGURE 3:**
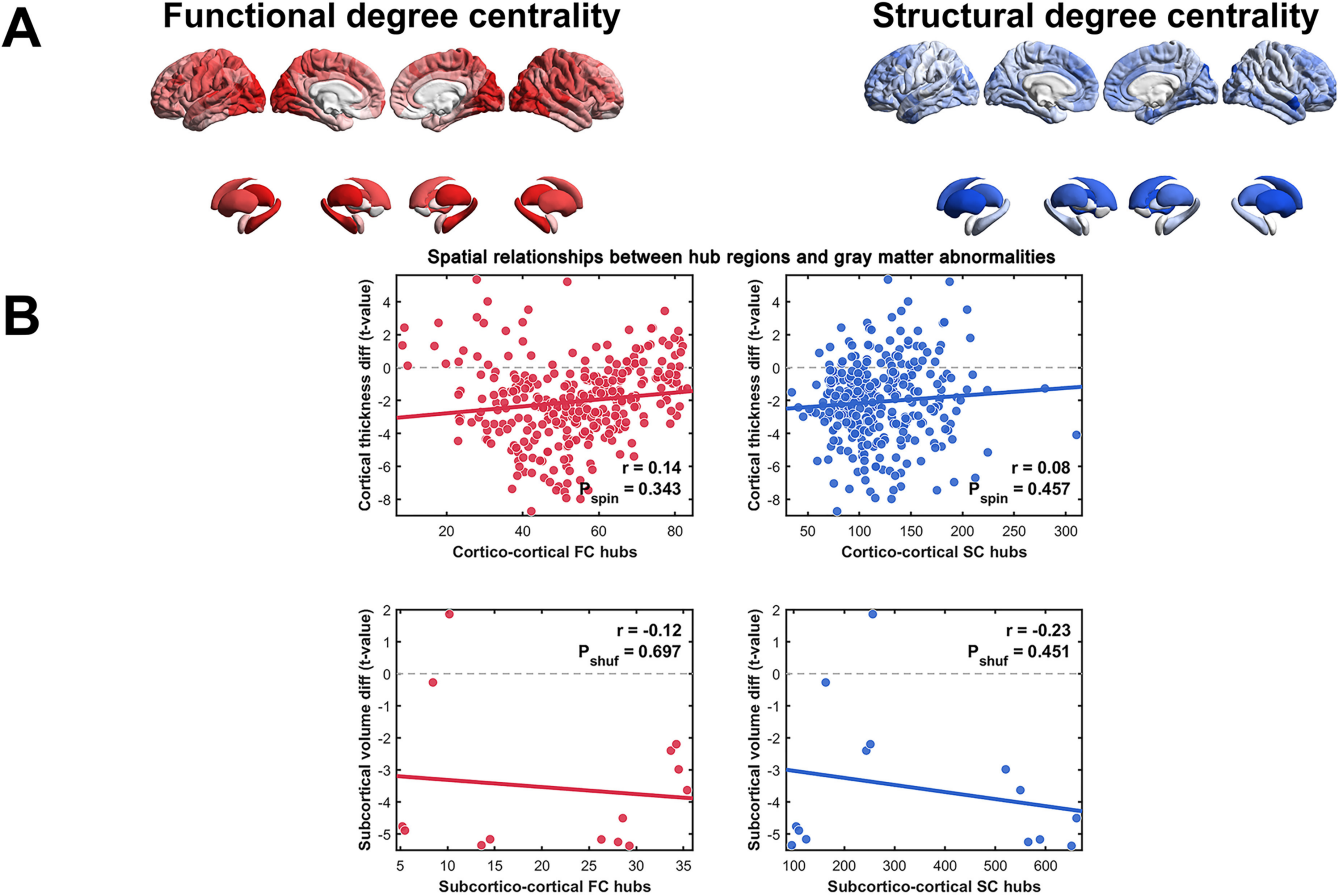
HE-related gray matter abnormalities do not preferentially localize to normative network hubs. **(A)** Normative weighted-degree maps derived from the HCP dataset are shown for FC (red) and SC (blue) networks; darker colors indicate higher degree and stronger hub-like topology. **(B)** Scatter plots show spatial correlations between signed HE gray matter abnormality values and normative hub maps. Negative abnormality values indicate lower gray matter measures in HE relative to HC and positive values indicate higher values; positive or negative correlations indicate whether higher-degree regions tend to show higher or lower signed abnormality values. Statistical significance was assessed with spin permutation tests for cortical analyses and random label-shuffling tests for subcortical analyses. HE, hepatic encephalopathy; HCP, Human Connectome Project; FC, functional connectivity; SC, structural connectivity.

After defining the HE gray matter abnormality map, we tested whether its spatial distribution was related to hub topology in normative brain networks. FC and SC matrices were generated from the HCP healthy adult sample, and weighted degree was calculated for each region (Figure 3A). Spatial correlations between the HE abnormality map and normative hub maps were not significant at either the cortical or subcortical level (all *P* > 0.05; Figure 3B). Thus, HE-related gray matter abnormalities did not follow a hub-preferential pattern. Hub maps obtained with the Schaefer400 parcellation were consistent with those derived from the DK308 and remained non-significant (Supplementary Figure 2 and Supplementary Table 4).

### 3.4 Cortical abnormalities in HE are primarily constrained by the SC network

**FIGURE 4:**
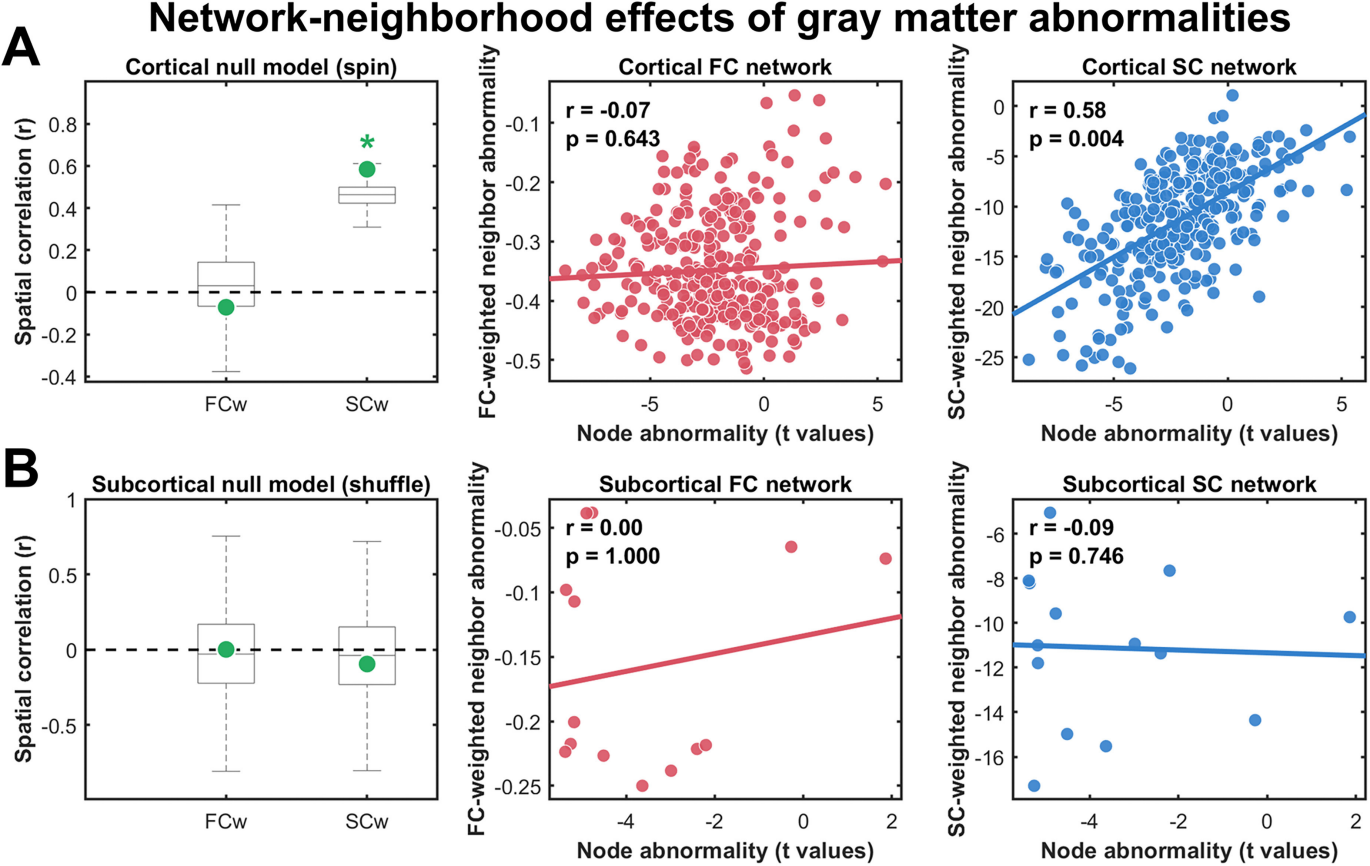
Cortical gray matter abnormalities in HE are preferentially related to structural network neighborhoods. Observed Spearman correlations between regional gray matter abnormality and the mean abnormality of connected neighbors were assessed separately for cortical and subcortical systems. **(A)** Cortical network-neighborhood analysis. Box plots show null distributions generated by spin permutation tests for FC-weighted and SC-weighted neighbor abnormality, and scatter plots show empirical associations between regional cortical abnormality and neighbor abnormality. The cortical SC-weighted association was significant, whereas the FC-weighted association was not. **(B)** Subcortical network-neighborhood analysis. Random label-shuffling null distributions and empirical scatter plots showed no significant FC- or SC-weighted subcortical associations. Across both panels, negative signed abnormality values indicate lower gray matter measures in HE relative to HC and positive values indicate higher values; positive correlations indicate that regions and their connected neighbors tend to show abnormality in the same direction, whereas negative correlations indicate opposite directions. HE, hepatic encephalopathy; FC, functional connectivity; SC, structural connectivity; HC, healthy controls. *Indicates statistical significance (*P* < 0.05).

We further analyzed the relationship between the gray matter abnormality burden of each brain region and the mean abnormality burden of its connected neighbors. At the cortical level (Figure 4A), regional abnormalities were significantly positively correlated with the mean abnormality burden of structurally connected neighbors (*r* = 0.58, P_*spin*_ = 0.004), with no significant correlation observed for functionally connected neighbors. Null-model analysis indicated that only the correlation from the SC-weighted analysis exceeded the random distribution. At the subcortical level (Figure 4B), no significant correlation was observed between neighbor and local abnormality burdens, regardless of functional or structural connectivity. These results indicate that HE-related gray matter abnormalities exhibit network dependence primarily at the level of cortical SC. SC-constrained neighborhood effects were also observed using the Schaefer400 parcellation, consistent with the DK308 results as supported by quantitative sensitivity metrics (Supplementary Figure 3 and Supplementary Table 4).

### 3.5 Group-level disease epicenter localization in HE

**FIGURE 5:**
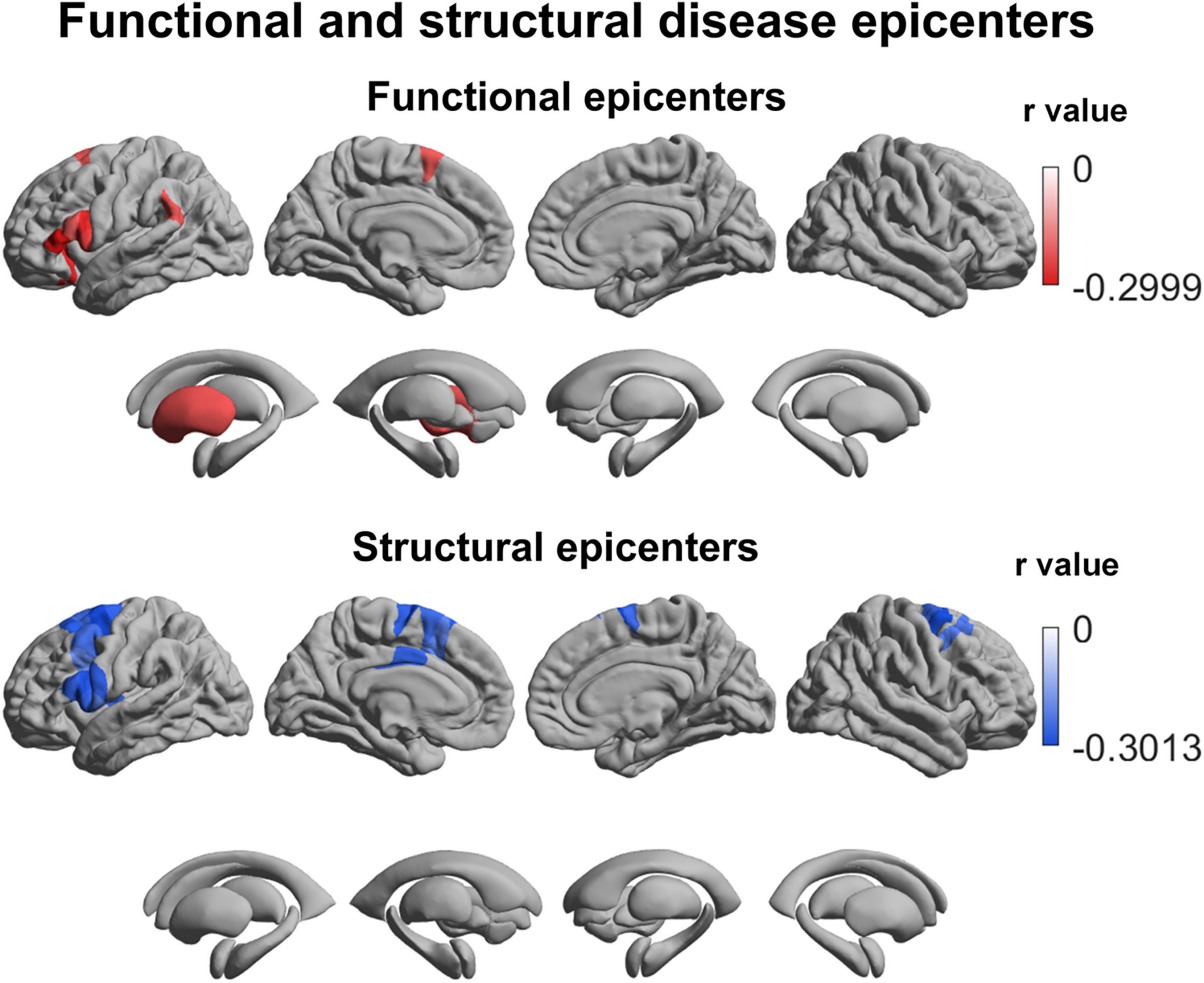
Functional and structural disease epicenters in HE. Brain maps show candidate disease epicenters identified from HCP normative connectivity profiles. The upper panel (red) shows functional epicenters, and the lower panel (blue) shows structural epicenters. For each seed region, its FC or SC profile was spatially correlated with the whole-brain signed HE gray matter abnormality map. The color scales show negative correlation coefficients, with more negative values indicating stronger inverse spatial associations between the seed connectivity profile and the signed HE gray matter abnormality map. Functional epicenters were mainly located in the left inferior frontal gyrus, lateral orbitofrontal cortex, and putamen, whereas structural epicenters were concentrated in bilateral superior frontal regions and in left inferior frontal, precentral, posterior cingulate, and insular cortices. HE, hepatic encephalopathy; HCP, Human Connectome Project; FC, functional connectivity; SC, structural connectivity.

After confirming that cortical abnormalities in HE are primarily related to SC, we conducted disease epicenter localization analysis (Figure 5). Group-level candidate disease epicenters were identified by correlating each region’s connectivity profile with the whole-brain HE gray matter abnormality map, with spin permutation testing used for significance assessment. FC analysis revealed that the top five significant epicenters were located in the triangular and opercular parts of the left inferior frontal gyrus, the left lateral orbitofrontal cortex, and the left putamen (Supplementary Table 2), indicating that functional-connectome-derived epicenters were concentrated in the left inferior frontal–orbitofrontal–striatum network. SC analysis showed that the top five significant disease epicenters were mainly located in the bilateral superior frontal regions and the opercular part of the left inferior frontal gyrus (Supplementary Table 3), suggesting that structural-connectome-derived disease epicenters were mainly concentrated in the bilateral superior frontal–left inferior frontal gyrus network. Overall, group-level HE disease epicenters were distributed in networks centered on the prefrontal cortex and including portions of the striatum.

### 3.6 Individual-level network model analysis

**FIGURE 6:**
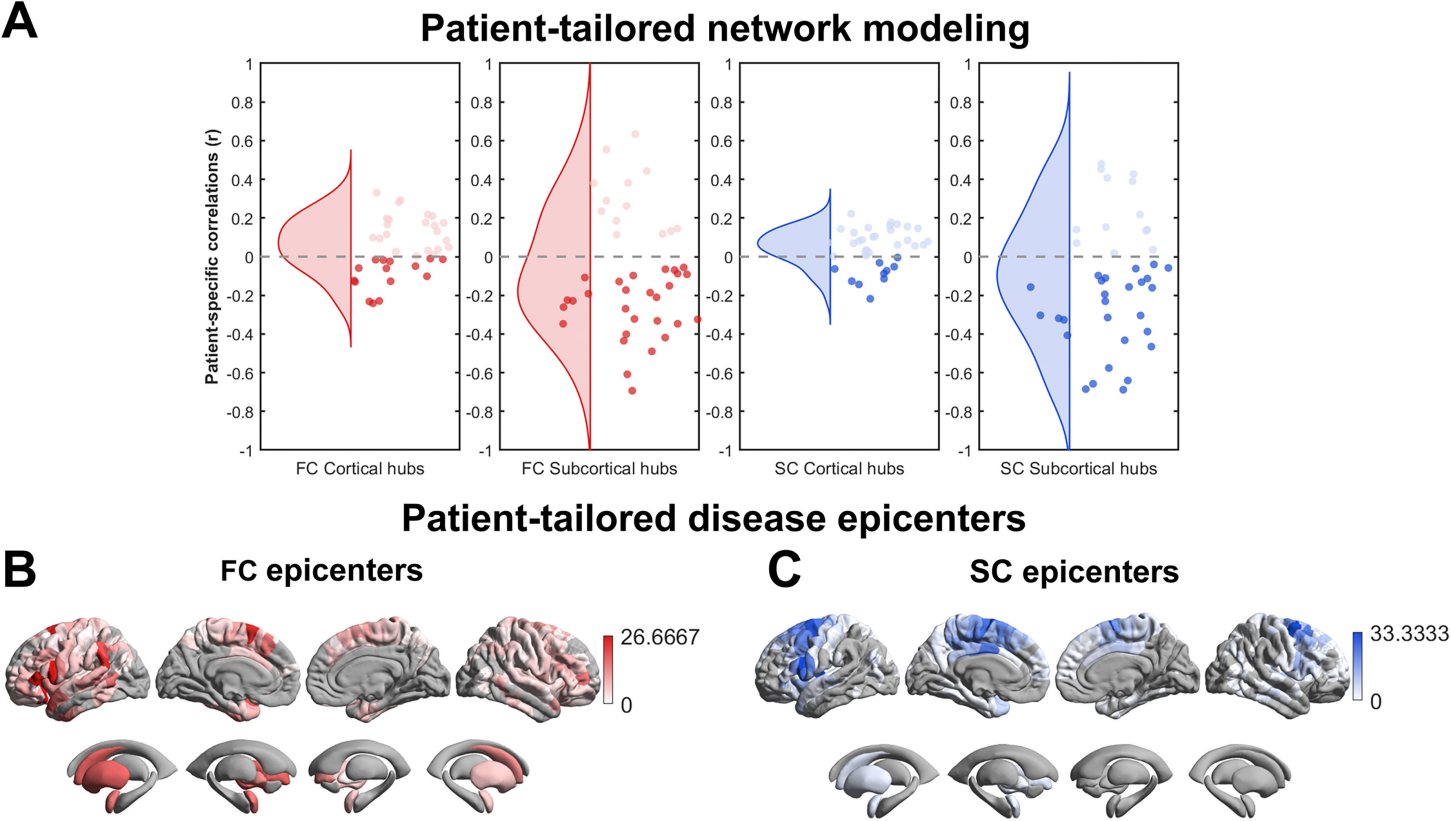
Patient-tailored network modeling in HE. **(A)** Violin and scatter plots show patient-specific associations between normative degree centrality and individualized abnormality maps across functional and structural cortical and subcortical networks. The dashed line indicates *r* = 0. **(B)** Spatial overlap of patient-specific candidate functional disease epicenters across the HE cohort. Color intensity indicates hit rate, defined as the percentage of patients in whom a region was identified as a significant functional epicenter. The highest functional hit rates were observed in the left lateral orbitofrontal cortex and left inferior frontal opercular and triangular subregions, with a maximum of 26.7%. (C) Spatial overlap of patient-specific candidate structural disease epicenters across the HE cohort. Color intensity indicates the hit rate of significant structural epicenters. The highest structural hit rates were observed in the left inferior frontal opercular subregion, left insula, and right superior frontal subregion, with a maximum of 33.3%. HE, hepatic encephalopathy; FC, functional connectivity; SC, structural connectivity.

At the individual level, we further analyzed the relationship between each patient’s individualized abnormality map and the topology of the normative brain network. Individual-level hub-related effects were generally weak, with correlation coefficients near zero across all models. Among the four models (Figure 6A), only the SC subcortical model showed a significant negative shift, with a median correlation of –0.111 and 60% of patients exhibiting negative correlations (*P* = 0.040). In contrast, individualized candidate disease epicenter analysis showed heterogeneous patient-level patterns with partial recurrence in prefrontal-related regions. At the FC level, regions with the highest hit rates were the left lateral orbitofrontal cortex and subregions of the opercular and triangular parts of the left inferior frontal gyrus, each with a hit rate of 26.7% (Figure 6B). At the SC level, the regions with the highest hit rates were subregions encompassing the opercular part of the left inferior frontal gyrus, the left insula, and the right superior frontal gyrus, each with a hit rate of 33.3% (Figure 6C). Overall, individual-level disease epicenters showed partial network-level recurrence rather than uniform single-region reproducibility, with recurrent regions partially overlapping group-level epicenters.

## 4 Discussion

This study combined group-level gray matter analysis with connectome modeling to characterize the whole-brain spatial organization of gray matter abnormalities observed in patients with HE. The principal findings were threefold. First, patients with HE exhibited widespread cortical and subcortical gray matter abnormalities, involving the prefrontal cortex, motor-related cortices, temporal lobe, insula, limbic system, basal ganglia, and thalamus. Second, these abnormalities did not exhibit a typical hub-preferential involvement pattern; however, at the cortical level, they were significantly coupled with the abnormality burden within SC neighborhoods, suggesting that their spatial organization is more strongly constrained by white matter connectivity architecture. Third, group-level disease epicenters were mainly concentrated in networks involving the left inferior frontal gyrus–orbitofrontal cortex–striatum and bilateral superior frontal regions–left inferior frontal gyrus. Although individual-level epicenters were heterogeneous, recurrent regions partially converged around these networks. Taken together, brain structural abnormalities observed in patients with HE are not only widespread but also exhibit identifiable features of network organization, suggesting that the observed gray matter abnormality pattern may reflect systems-level involvement across cortical–subcortical circuits.

Group-level gray matter analysis showed that HE-related gray matter abnormalities were mainly distributed across the prefrontal cortex, motor-related cortices, temporal lobe, limbic system, basal ganglia, and thalamus. This pattern suggests that gray matter abnormalities observed in patients with HE are not confined to a single region but involve multiple functional systems related to cognitive control, attentional regulation, psychomotor function, and cortical–subcortical integration. Given the common clinical manifestations of HE, including attentional deficits, impaired executive function, and psychomotor slowing (23), this abnormality pattern is highly consistent with its clinical phenotype at the functional systems level. Previous studies have reported that HE is associated with widespread abnormalities in both cortical and subcortical gray matter (24). Studies of minimal HE also reported abnormal gray matter structural covariance network topology, impaired interhemispheric coordination, and altered thalamic structural connectivity (25), indicating that HE-related brain alterations extend beyond local anatomical structures to broader network reorganization. Consistent with these findings, our study further indicates that this widespread involvement is not merely a co-occurrence of abnormal regions or networks but exhibits an identifiable connectome-based spatial organization across the whole brain.

Further connectome analyses showed no significant spatial correlations between HE-related gray matter abnormalities and the distributions of normative functional or structural hubs, suggesting that these gray matter abnormalities do not conform to a typical hub-preferential involvement pattern. At the cortical level, regional abnormalities were significantly positively correlated with the mean abnormality burden of structurally connected neighbors, whereas no significant correlation was observed for functionally connected neighbors. These findings suggest that cortical abnormality distribution in HE is primarily constrained by SC. This pattern may reflect the pathophysiological characteristics of HE. The onset and progression of HE are mainly associated with diffuse pathological processes, including hyperammonemia, neuroinflammation, astrocytic dysfunction, and cerebral edema (26, 27). These factors are more likely to exert widespread effects across brain systems rather than selectively affecting a small number of highly connected nodes. Consequently, macroscale gray matter abnormalities in HE may not preferentially involve highly connected hubs but instead form a coordinated distribution across anatomically interconnected regions. Moreover, SC reflects a stable white matter scaffold, whereas FC is more susceptible to fluctuations in metabolic state, neural activity reorganization, and compensatory mechanisms. Therefore, in characterizing the spatial organization of chronic gray matter abnormalities, SC may provide a more stable and consistent framework than FC. Overall, HE-related connectome dependence is primarily reflected in the constraints imposed by SC on the spatial distribution of abnormalities, rather than in preferential hub involvement.

Disease epicenter analysis further identified candidate connectivity anchors associated with the distribution of gray matter abnormalities observed in patients with HE from a connectome perspective. The group-level results showed that functional-connectome-derived disease epicenters were mainly concentrated in networks involving the left inferior frontal gyrus, orbitofrontal cortex, and striatum, whereas structural-connectome-derived disease epicenters were mainly concentrated in networks involving the bilateral superior frontal regions and left inferior frontal gyrus. In individual-level analyses, regions including the left inferior frontal gyrus, left lateral orbitofrontal cortex, left insula, and right superior frontal gyrus were also identified with relatively high recurrence rates. It should be emphasized that disease epicenters are not equivalent to the true sites of pathological onset or propagation; rather, they refer to key nodes whose normative connectivity profiles best explain the spatial distribution of whole-brain gray matter abnormalities. This interpretive framework is consistent with the definition used in previous connectome-based disease epicenter studies (22). Therefore, candidate epicenters identified here are best interpreted as exploratory connectivity anchors within the HE gray matter abnormality network, rather than fixed “sites of onset.” The distribution of these epicenter regions is broadly consistent with previous neuroimaging evidence of HE-related brain injury. Previous studies have reported that, during progression from no HE to minimal HE, the prefrontal cortex and circuits involving the caudate, putamen, and thalamus show progressive abnormalities (28), suggesting that the prefrontal–striatal–thalamic system is a key network affected early in HE. Consistent with this evidence, the inferior frontal gyrus, superior frontal regions, and orbitofrontal cortex were repeatedly identified in the group- and individual-level epicenter analyses in the present study, suggesting that the prefrontal control system may play a key organizing role in the whole-brain distribution of gray matter abnormalities in HE. The subcortical epicenter findings also have plausible disease-specific relevance. The basal ganglia are classically vulnerable in chronic liver disease-related brain injury, with abnormalities potentially linked to manganese deposition and neurotoxic effects (29). The thalamus serves as a key relay for cortical–subcortical information integration and arousal regulation. Previous studies show that patients with minimal HE already exhibit abnormal SC between the thalamus and cortical/subcortical regions, which may be associated with cognitive dysfunction (30). Therefore, the inferior frontal gyrus–orbitofrontal cortex–striatum and superior frontal–inferior frontal gyrus epicenter networks identified in this study may reflect a network-level injury pattern involving both the prefrontal control system and cortical–striatal–thalamic circuits in the metabolic and toxic milieu of HE. In addition, the repeated identification of the insula at the individual level suggests that HE-related gray matter abnormalities may also involve salience network nodes. A previous voxel-level meta-analysis of gray matter alterations in HE and minimal HE showed that the insula, basal ganglia, and anterior cingulate gyrus are relatively consistent sites of gray matter abnormalities (31). The insula is closely connected with the prefrontal control network and cortical–subcortical circuits and is involved in interoceptive integration, salience detection, and cognitive control switching. Therefore, the repeated appearance of the insula among individual-level epicenters suggests that, despite interindividual heterogeneity, HE-related abnormalities are organized around the prefrontal control system, cortical–striatal–thalamic circuits, and insula-related networks. Overall, disease epicenter analysis complements network neighborhood analysis and supports the view that gray matter abnormalities observed in patients with HE are not merely a collection of changes in isolated regions, but represent a cross-network distribution pattern shaped by connectome architecture.

Several limitations should be acknowledged. First, this study used a cross-sectional design, which precludes direct inference of the dynamic evolution, pathological onset, or propagation of HE-related gray matter abnormalities. Accordingly, the present findings should be considered hypothesis-generating and cannot establish early-detection biomarkers or therapeutic targets without validation in longitudinal and clinically characterized cohorts. Second, the sample size was relatively limited, and patients with HE may exhibit substantial heterogeneity in disease stage, precipitating factors, metabolic status, and treatment history, potentially affecting the stability of individual-level network model results. Third, this study used a normative connectome rather than patient-specific connectomes as the reference. Although this approach provides a healthy reference framework for testing whether HE-related morphometric abnormalities are aligned with canonical connectome architecture, the HCP sample consisted of younger healthy adults and may not fully match the HE cohort. Normative connectomes also cannot capture patient-specific connectivity remodeling. Future studies incorporating resting-state functional MRI and diffusion MRI from the same HE patients are needed to validate these findings at the individual connectome level. Fourth, this study did not include a cirrhotic non-HE control group. Therefore, the observed gray matter abnormalities cannot be attributed exclusively to HE itself and may partly reflect cirrhosis, chronic liver disease, systemic inflammation, medication effects, nutritional status, or other liver disease-related confounders. Future studies including cirrhotic patients without HE are needed to better distinguish HE-specific effects from cirrhosis-related brain changes.

## 5 Conclusion

In summary, this study demonstrates that gray matter abnormalities observed in patients with HE exhibit a widespread yet non-random whole-brain spatial organization pattern. This pattern did not display typical hub-preferential vulnerability; instead, it was primarily characterized by cortical abnormalities constrained by SC and associated with prefrontal-centered disease epicenter networks. Although individual-level network patterns were heterogeneous, disease epicenters showed partial spatial recurrence. These findings suggest that gray matter abnormalities observed in patients with HE are best understood as a cross-network spatial pattern associated with connectome architecture, providing new insights into the neuroimaging features of HE and generating hypotheses for future longitudinal and clinically validated studies.

## Data Availability

The original contributions presented in this study are included in the article/Supplementary material, further inquiries can be directed to the corresponding authors.
