# Supplementary material for: Connectome-based mapping of gray matter abnormalities in hepatic encephalopathy
Source: Front Med (Lausanne). 2026 Jul 10;13:1886303. doi: 10.3389/fmed.2026.1886303 (PMC13395610; doi:10.3389/fmed.2026.1886303)
Supplement: Supplementary file 1 [file Table_1.docx]

**Connectome-based mapping of gray matter abnormalities in hepatic encephalopathy**

**Supplementary Material**

# 1 Supplementary Methods

# **1.1 MRI acquisition parameters**

# T1-weighted MRI was acquired using a three-dimensional sagittal sequence with the following parameters: repetition time = 7 ms, echo time = 3 ms, inversion time = 750 ms, flip angle = 9°, field of view = 256 × 256 mm², matrix = 256 × 256, voxel size = 1.0 × 1.0 × 1.0 mm³, no interslice gap, and 176 slices.

# **1.2 Spatial permutation and sensitivity analyses**

Cortical spin permutation was performed by projecting regional coordinates onto a spherical surface and randomly rotating the sphere. After each rotation, regional values were reassigned to the nearest brain regions, generating a spatially constrained null distribution for map-level correlations (1). For subcortical regions, region labels were randomly shuffled while preserving the number of regions (2). The Schaefer400 sensitivity analyses repeated the group-level morphological comparison, hub-related analysis, and network-neighborhood analysis with the alternative cortical parcellation. Quantitative consistency metrics for these sensitivity analyses, including the number and direction of FDR-significant regions, hub-related effect sizes, and network-neighborhood effect sizes with permutation-based P values, are summarized in Supplementary Table S4.

# **1.3 HCP preprocessing and connectome reconstruction**

HCP resting-state fMRI preprocessing included distortion correction, head-motion correction, bias-field correction, brain tissue extraction, intensity normalization, registration to standard space, and FIX-based removal of noise components (3, 4). Diffusion MRI preprocessing included b0 intensity normalization, susceptibility distortion correction, eddy-current correction, and motion correction. Regional connectivity matrices were reconstructed using the same cortical and subcortical parcellation scheme as in the HE analysis. For FC, pairwise Pearson correlations were calculated among regional resting-state fMRI time series. Negative correlations were set to zero, and individual matrices were Fisher z-transformed before averaging across HCP participants.

For SC, anatomically constrained tractography was performed using tissue types derived from T1-weighted segmentation. Multi-shell multi-tissue response functions were estimated, followed by constrained spherical deconvolution and intensity normalization. The initial tractogram included 40 million streamlines, with a maximum streamline length of 250 and a fractional anisotropy threshold of 0.06. SIFT2 was applied to assign weights to streamlines. Reconstructed streamlines were mapped to the 308 cortical and 14 subcortical regions to generate subject-level SC matrices. For each pair of regions, SC strength was defined as the SIFT2-weighted streamline-based connectivity between the two regions (5). A distance-dependent thresholding procedure was applied to construct the group-level SC matrix while preserving edge-length distributions. SC weights were then log-transformed to reduce connectivity strength variance. Thus, the SC weights used in the main analyses represented log-transformed SIFT2-weighted SC values rather than raw streamline counts.

**1.4 Standard weight-normalized sensitivity analysis.**

To test whether the network-neighborhood findings depended on the normalization scheme, we repeated the network-neighborhood analysis using a standard weight-normalized average:

$\text{(}\text{A}_{\text{i}}^{\text{weig}\text{h}\text{ted}}\text{=}\frac{\sum_{\text{j}\text{=1}}^{\text{N}_{\text{i}}} \text{a}_{\text{j}}\text{W}_{\text{ij}}}{\sum_{\text{j}\text{=1}}^{\text{N}_{\text{i}}} \text{W}_{\text{ij}}}\text{, }\text{j}\text{≠}\text{i}\text{)}$.

Spearman correlations were then computed between regional gray matter abnormality and weight-normalized neighbor abnormality, with spin permutation used for cortical regions and random label permutation used for subcortical regions. Using the standard weight-normalized formulation, the cortical SC-weighted association remained significant (r = 0.66, P_spin_ < 0.001). The cortical FC-weighted association also reached significance (r = 0.49, P_spin_ < 0.001). In contrast, subcortical FC- and SC-weighted associations remained nonsignificant (FC: r = 0.21, P_shuf_ = 0.473; SC: r = −0.11, P_shuf_ = 0.725).

# **1.5 Individual-level epicenter recurrence**

Individual-level disease epicenters were identified by correlating each patient's individualized abnormality map with normative FC and SC seed-connectivity profiles for all regions. Cortical significance was assessed with 1,000 spin permutations, with corresponding label-permutation tests used for subcortical maps. For each region, recurrence was summarized as the hit rate, defined as the percentage of patients in whom that region reached significance as a functional or structural epicenter.

# **2 Supplementary Figures and Tables**

# **2.1 Supplementary Figures**

**
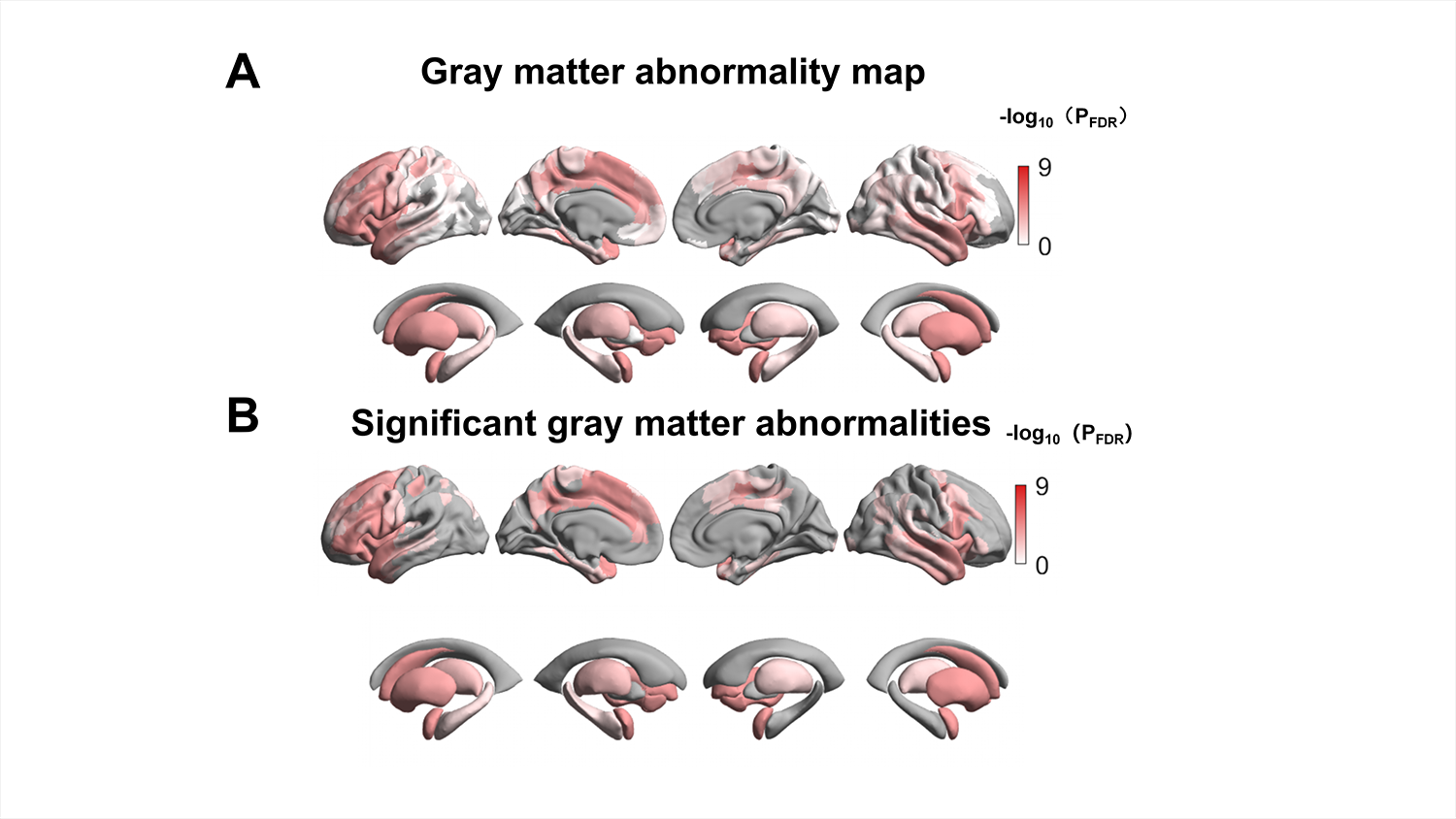
Supplementary Figure 1 HE is associated with widespread cortical and subcortical gray matter abnormalities.**

**(A)** Unthresholded group-level statistical maps showing cortical thickness and subcortical volume differences between HE and HC. Cortical maps are displayed on the brain surface, and subcortical maps are shown separately.

**(B)** Regions surviving FDR correction. Color bars indicate −log10(P_FDR_), with higher values indicating stronger statistical evidence for group differences.

Abbreviations: HE = hepatic encephalopathy; HC = healthy controls; FDR = false discovery rate.

**
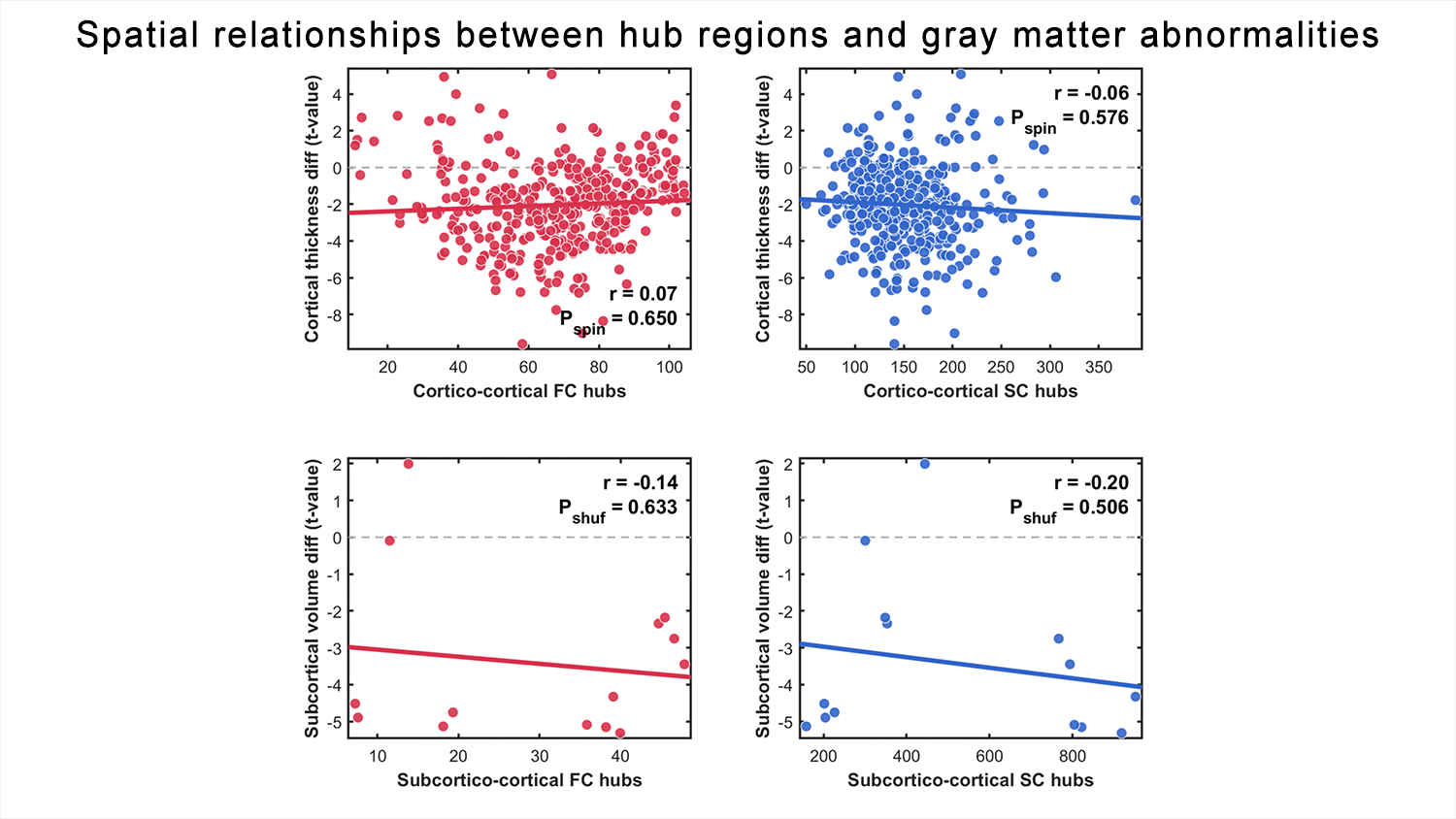
**

**Supplementary Figure 2 HE-related gray matter abnormalities do not preferentially localize to normative network hubs.**

Scatter plots show spatial correlations between signed HE gray matter abnormality values and normative hub maps. Negative abnormality values indicate lower gray matter measures in HE relative to HC and positive values indicate higher values; positive or negative correlations indicate whether higher-degree regions tend to show higher or lower signed abnormality values. Statistical significance was assessed with spin permutation tests for cortical analyses and random label-shuffling tests for subcortical analyses.

Abbreviations: HE = hepatic encephalopathy; HCP = Human Connectome Project; FC = functional connectivity; SC = structural connectivity.


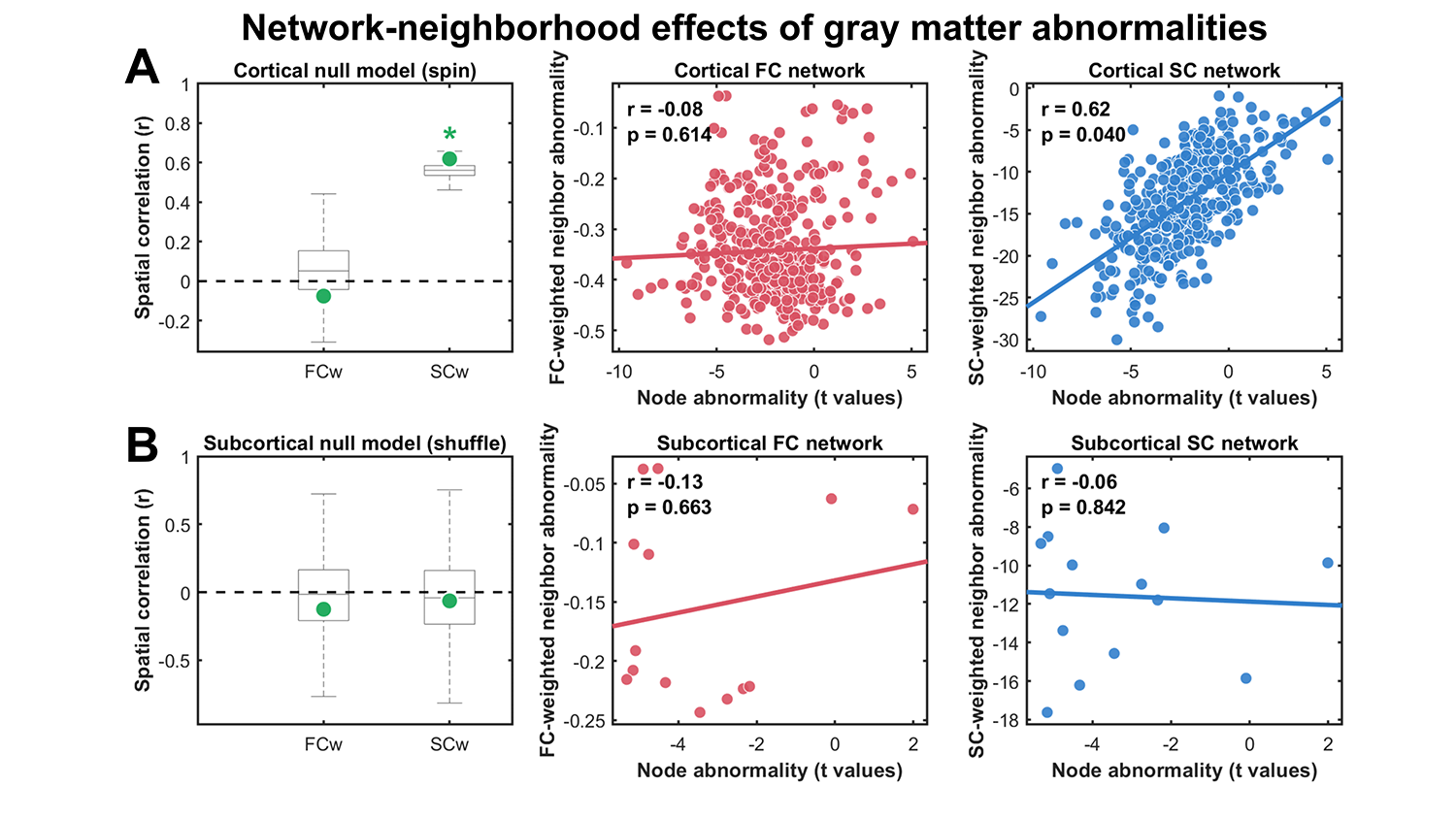


**Supplementary Figure 3** **Cortical abnormalities in HE are preferentially related to structural network neighborhoods.**

Observed Spearman correlations between regional abnormality and the mean abnormality of connected neighbors were assessed separately for cortical and subcortical systems.

1. Cortical network-neighborhood analysis. Box plots show null distributions generated by spin permutation tests for FC-weighted and SC-weighted neighbor abnormality. Scatter plots show empirical associations between regional cortical abnormality and FC-weighted or SC-weighted neighbor abnormality. In the cortex, regional abnormality was significantly associated with SC-weighted, but not FC-weighted, neighbor abnormality.

**(B)** Subcortical network-neighborhood analysis. Box plots show null distributions generated by random label-shuffling tests, and scatter plots show empirical associations between regional subcortical abnormality and FC-weighted or SC-weighted neighbor abnormality. No significant associations were observed in subcortical regions.
Abbreviations: HE = hepatic encephalopathy; FC = functional connectivity; SC = structural connectivity.

# 2.2 Supplementary Tables

**Supplementary Table 1. Brain regions showing significant cortical thickness or subcortical volume differences between the HE and HC groups after FDR correction**

| **Region ID** | **Region Name** | **Region Type** | **t value** | **uncorrected p value** | **P_FDR_** | **Effect Direction** |
| --- | --- | --- | --- | --- | --- | --- |
| 65 | lh_parsopercularis_part2 | Cortical | -8.728 | 1.761E-13 | 5.670E-11 | HE < HC |
| 114 | lh_superiorfrontal_part6 | Cortical | -7.982 | 5.742E-12 | 8.047E-10 | HE < HC |
| 239 | rh_precentral_part1 | Cortical | -7.925 | 7.497E-12 | 8.047E-10 | HE < HC |
| 135 | lh_superiortemporal_part4 | Cortical | -7.543 | 4.401E-11 | 3.543E-09 | HE < HC |
| 118 | lh_superiorfrontal_part10 | Cortical | -7.445 | 6.901E-11 | 3.703E-09 | HE < HC |
| 121 | lh_superiorfrontal_part13 | Cortical | -7.453 | 6.663E-11 | 3.703E-09 | HE < HC |
| 69 | lh_parstriangularis_part2 | Cortical | -7.373 | 9.644E-11 | 4.436E-09 | HE < HC |
| 66 | lh_parsopercularis_part3 | Cortical | -7.222 | 1.922E-10 | 7.737E-09 | HE < HC |
| 80 | lh_posteriorcingulate_part1 | Cortical | -7.039 | 4.446E-10 | 1.591E-08 | HE < HC |
| 138 | lh_superiortemporal_part7 | Cortical | -6.959 | 6.397E-10 | 2.060E-08 | HE < HC |
| 83 | lh_precentral_part2 | Cortical | -6.846 | 1.064E-09 | 3.115E-08 | HE < HC |
| 115 | lh_superiorfrontal_part7 | Cortical | -6.699 | 2.065E-09 | 5.540E-08 | HE < HC |
| 308 | rh_insula_part4 | Cortical | -6.568 | 3.712E-09 | 9.194E-08 | HE < HC |
| 116 | lh_superiorfrontal_part8 | Cortical | -6.401 | 7.835E-09 | 1.802E-07 | HE < HC |
| 44 | lh_lateralorbitofrontal_part4 | Cortical | -6.341 | 1.021E-08 | 2.193E-07 | HE < HC |
| 219 | rh_parsopercularis_part1 | Cortical | -6.278 | 1.348E-08 | 2.713E-07 | HE < HC |
| 221 | rh_parsopercularis_part3 | Cortical | -6.239 | 1.601E-08 | 3.033E-07 | HE < HC |
| 85 | lh_precentral_part4 | Cortical | -6.204 | 1.867E-08 | 3.339E-07 | HE < HC |
| 305 | rh_insula_part1 | Cortical | -6.020 | 4.161E-08 | 7.052E-07 | HE < HC |
| 110 | lh_superiorfrontal_part2 | Cortical | -5.997 | 4.596E-08 | 7.399E-07 | HE < HC |
| 5 | lh_caudalmiddlefrontal_part2 | Cortical | -5.838 | 9.131E-08 | 1.400E-06 | HE < HC |
| 67 | lh_parsorbitalis_part1 | Cortical | -5.683 | 1.770E-07 | 2.589E-06 | HE < HC |
| 133 | lh_superiortemporal_part2 | Cortical | -5.673 | 1.849E-07 | 2.589E-06 | HE < HC |
| 152 | lh_insula_part4 | Cortical | -5.656 | 1.987E-07 | 2.666E-06 | HE < HC |
| 294 | rh_superiortemporal_part6 | Cortical | -5.591 | 2.617E-07 | 3.371E-06 | HE < HC |
| 292 | rh_superiortemporal_part4 | Cortical | -5.516 | 3.589E-07 | 4.444E-06 | HE < HC |
| 64 | lh_parsopercularis_part1 | Cortical | -5.489 | 4.019E-07 | 4.793E-06 | HE < HC |
| 289 | rh_superiortemporal_part1 | Cortical | -5.467 | 4.404E-07 | 5.065E-06 | HE < HC |
| 89 | lh_precentral_part8 | Cortical | -5.397 | 5.901E-07 | 6.552E-06 | HE < HC |
| 321 | Right-Putamen | Subcortical | -5.374 | 6.494E-07 | 6.971E-06 | HE < HC |
| 317 | Right-Amygdala | Subcortical | -5.355 | 7.021E-07 | 7.293E-06 | HE < HC |
| 256 | rh_rostralmiddlefrontal_part1 | Cortical | 5.347 | 7.253E-07 | 7.298E-06 | HE > HC |
| 94 | lh_precuneus_part4 | Cortical | -5.274 | 9.833E-07 | 9.595E-06 | HE < HC |
| 311 | Left-Caudate | Subcortical | -5.253 | 1.072E-06 | 1.015E-05 | HE < HC |
| 257 | rh_rostralmiddlefrontal_part2 | Cortical | 5.214 | 1.254E-06 | 1.154E-05 | HE > HC |
| 310 | Left-Amygdala | Subcortical | -5.168 | 1.519E-06 | 1.322E-05 | HE < HC |
| 318 | Right-Caudate | Subcortical | -5.174 | 1.481E-06 | 1.322E-05 | HE < HC |
| 120 | lh_superiorfrontal_part12 | Cortical | -5.150 | 1.634E-06 | 1.385E-05 | HE < HC |
| 68 | lh_parstriangularis_part1 | Cortical | -5.119 | 1.848E-06 | 1.526E-05 | HE < HC |
| 62 | lh_paracentral_part2 | Cortical | -5.081 | 2.157E-06 | 1.737E-05 | HE < HC |
| 43 | lh_lateralorbitofrontal_part3 | Cortical | -5.020 | 2.758E-06 | 2.166E-05 | HE < HC |
| 316 | Right-Accumbens-area | Subcortical | -4.894 | 4.563E-06 | 3.498E-05 | HE < HC |
| 150 | lh_insula_part2 | Cortical | -4.873 | 4.972E-06 | 3.723E-05 | HE < HC |
| 4 | lh_caudalmiddlefrontal_part1 | Cortical | -4.805 | 6.489E-06 | 4.749E-05 | HE < HC |
| 6 | lh_caudalmiddlefrontal_part3 | Cortical | -4.792 | 6.846E-06 | 4.792E-05 | HE < HC |
| 7 | lh_caudalmiddlefrontal_part4 | Cortical | -4.796 | 6.730E-06 | 4.792E-05 | HE < HC |
| 156 | rh_caudalmiddlefrontal_part1 | Cortical | -4.767 | 7.549E-06 | 5.064E-05 | HE < HC |
| 309 | Left-Accumbens-area | Subcortical | -4.767 | 7.531E-06 | 5.064E-05 | HE < HC |
| 111 | lh_superiorfrontal_part3 | Cortical | -4.727 | 8.840E-06 | 5.809E-05 | HE < HC |
| 241 | rh_precentral_part3 | Cortical | -4.688 | 1.026E-05 | 6.606E-05 | HE < HC |
| 272 | rh_superiorfrontal_part7 | Cortical | -4.652 | 1.183E-05 | 7.468E-05 | HE < HC |
| 179 | rh_inferiortemporal_part1 | Cortical | -4.617 | 1.352E-05 | 8.369E-05 | HE < HC |
| 103 | lh_rostralmiddlefrontal_part5 | Cortical | -4.595 | 1.471E-05 | 8.938E-05 | HE < HC |
| 314 | Left-Putamen | Subcortical | -4.509 | 2.043E-05 | 1.218E-04 | HE < HC |
| 197 | rh_lateralorbitofrontal_part3 | Cortical | -4.503 | 2.090E-05 | 1.224E-04 | HE < HC |
| 147 | lh_temporalpole_part1 | Cortical | -4.457 | 2.489E-05 | 1.431E-04 | HE < HC |
| 154 | rh_bankssts_part2 | Cortical | -4.431 | 2.751E-05 | 1.527E-04 | HE < HC |
| 224 | rh_parstriangularis_part2 | Cortical | -4.432 | 2.734E-05 | 1.527E-04 | HE < HC |
| 119 | lh_superiorfrontal_part11 | Cortical | -4.413 | 2.944E-05 | 1.607E-04 | HE < HC |
| 266 | rh_superiorfrontal_part1 | Cortical | -4.408 | 2.999E-05 | 1.610E-04 | HE < HC |
| 60 | lh_parahippocampal_part2 | Cortical | -4.390 | 3.208E-05 | 1.694E-04 | HE < HC |
| 24 | lh_inferiortemporal_part1 | Cortical | -4.358 | 3.612E-05 | 1.876E-04 | HE < HC |
| 112 | lh_superiorfrontal_part4 | Cortical | -4.279 | 4.858E-05 | 2.483E-04 | HE < HC |
| 92 | lh_precuneus_part2 | Cortical | -4.259 | 5.230E-05 | 2.631E-04 | HE < HC |
| 86 | lh_precentral_part5 | Cortical | -4.237 | 5.671E-05 | 2.809E-04 | HE < HC |
| 291 | rh_superiortemporal_part3 | Cortical | -4.088 | 9.747E-05 | 4.755E-04 | HE < HC |
| 157 | rh_caudalmiddlefrontal_part2 | Cortical | -4.044 | 1.141E-04 | 5.484E-04 | HE < HC |
| 61 | lh_paracentral_part1 | Cortical | -4.034 | 1.182E-04 | 5.598E-04 | HE < HC |
| 245 | rh_precentral_part7 | Cortical | -4.015 | 1.268E-04 | 5.832E-04 | HE < HC |
| 267 | rh_superiorfrontal_part2 | Cortical | 4.018 | 1.251E-04 | 5.832E-04 | HE > HC |
| 208 | rh_middletemporal_part1 | Cortical | -3.919 | 1.782E-04 | 8.083E-04 | HE < HC |
| 117 | lh_superiorfrontal_part9 | Cortical | -3.908 | 1.847E-04 | 8.259E-04 | HE < HC |
| 260 | rh_rostralmiddlefrontal_part5 | Cortical | -3.890 | 1.968E-04 | 8.681E-04 | HE < HC |
| 108 | lh_rostralmiddlefrontal_part10 | Cortical | -3.815 | 2.558E-04 | 1.098E-03 | HE < HC |
| 185 | rh_isthmuscingulate_part2 | Cortical | -3.817 | 2.541E-04 | 1.098E-03 | HE < HC |
| 153 | rh_bankssts_part1 | Cortical | -3.808 | 2.618E-04 | 1.109E-03 | HE < HC |
| 137 | lh_superiortemporal_part6 | Cortical | -3.754 | 3.161E-04 | 1.322E-03 | HE < HC |
| 73 | lh_postcentral_part2 | Cortical | -3.742 | 3.285E-04 | 1.332E-03 | HE < HC |
| 88 | lh_precentral_part7 | Cortical | -3.747 | 3.234E-04 | 1.332E-03 | HE < HC |
| 209 | rh_middletemporal_part2 | Cortical | -3.740 | 3.310E-04 | 1.332E-03 | HE < HC |
| 220 | rh_parsopercularis_part2 | Cortical | -3.662 | 4.309E-04 | 1.713E-03 | HE < HC |
| 315 | Left-Thalamus | Subcortical | -3.634 | 4.740E-04 | 1.861E-03 | HE < HC |
| 247 | rh_precentral_part9 | Cortical | -3.606 | 5.215E-04 | 2.023E-03 | HE < HC |
| 230 | rh_postcentral_part2 | Cortical | -3.532 | 6.657E-04 | 2.552E-03 | HE < HC |
| 105 | lh_rostralmiddlefrontal_part7 | Cortical | -3.518 | 6.979E-04 | 2.613E-03 | HE < HC |
| 261 | rh_rostralmiddlefrontal_part6 | Cortical | 3.520 | 6.923E-04 | 2.613E-03 | HE > HC |
| 11 | lh_fusiform_part1 | Cortical | -3.465 | 8.289E-04 | 3.068E-03 | HE < HC |
| 242 | rh_precentral_part4 | Cortical | -3.451 | 8.688E-04 | 3.179E-03 | HE < HC |
| 162 | rh_cuneus_part3 | Cortical | 3.441 | 8.973E-04 | 3.246E-03 | HE > HC |
| 31 | lh_isthmuscingulate_part2 | Cortical | -3.432 | 9.237E-04 | 3.304E-03 | HE < HC |
| 56 | lh_middletemporal_part3 | Cortical | -3.429 | 9.337E-04 | 3.304E-03 | HE < HC |
| 13 | lh_fusiform_part3 | Cortical | -3.410 | 9.915E-04 | 3.416E-03 | HE < HC |
| 101 | lh_rostralmiddlefrontal_part3 | Cortical | -3.408 | 9.971E-04 | 3.416E-03 | HE < HC |
| 210 | rh_middletemporal_part3 | Cortical | -3.413 | 9.817E-04 | 3.416E-03 | HE < HC |
| 159 | rh_caudalmiddlefrontal_part4 | Cortical | -3.403 | 1.013E-03 | 3.433E-03 | HE < HC |
| 128 | lh_superiorparietal_part7 | Cortical | -3.387 | 1.069E-03 | 3.585E-03 | HE < HC |
| 211 | rh_middletemporal_part4 | Cortical | -3.373 | 1.116E-03 | 3.705E-03 | HE < HC |
| 97 | lh_precuneus_part7 | Cortical | -3.365 | 1.145E-03 | 3.763E-03 | HE < HC |
| 107 | lh_rostralmiddlefrontal_part9 | Cortical | -3.348 | 1.209E-03 | 3.931E-03 | HE < HC |
| 10 | lh_entorhinal_part1 | Cortical | -3.326 | 1.295E-03 | 4.089E-03 | HE < HC |
| 213 | rh_middletemporal_part6 | Cortical | -3.328 | 1.288E-03 | 4.089E-03 | HE < HC |
| 290 | rh_superiortemporal_part2 | Cortical | -3.326 | 1.295E-03 | 4.089E-03 | HE < HC |
| 297 | rh_supramarginal_part3 | Cortical | -3.291 | 1.451E-03 | 4.536E-03 | HE < HC |
| 301 | rh_supramarginal_part7 | Cortical | -3.262 | 1.586E-03 | 4.909E-03 | HE < HC |
| 303 | rh_temporalpole_part1 | Cortical | -3.255 | 1.624E-03 | 4.981E-03 | HE < HC |
| 178 | rh_inferiorparietal_part10 | Cortical | -3.162 | 2.166E-03 | 6.581E-03 | HE < HC |
| 238 | rh_posteriorcingulate_part2 | Cortical | -3.093 | 2.672E-03 | 8.008E-03 | HE < HC |
| 243 | rh_precentral_part5 | Cortical | -3.091 | 2.686E-03 | 8.008E-03 | HE < HC |
| 215 | rh_parahippocampal_part2 | Cortical | -3.076 | 2.816E-03 | 8.319E-03 | HE < HC |
| 113 | lh_superiorfrontal_part5 | Cortical | -3.057 | 2.974E-03 | 8.649E-03 | HE < HC |
| 302 | rh_frontalpole_part1 | Cortical | 3.057 | 2.982E-03 | 8.649E-03 | HE > HC |
| 21 | lh_inferiorparietal_part6 | Cortical | -3.049 | 3.054E-03 | 8.780E-03 | HE < HC |
| 59 | lh_parahippocampal_part1 | Cortical | -3.045 | 3.088E-03 | 8.801E-03 | HE < HC |
| 96 | lh_precuneus_part6 | Cortical | -3.038 | 3.157E-03 | 8.853E-03 | HE < HC |
| 218 | rh_paracentral_part3 | Cortical | -3.037 | 3.162E-03 | 8.853E-03 | HE < HC |
| 98 | lh_rostralanteriorcingulate_part1 | Cortical | -3.028 | 3.253E-03 | 9.031E-03 | HE < HC |
| 99 | lh_rostralmiddlefrontal_part1 | Cortical | -3.000 | 3.530E-03 | 9.715E-03 | HE < HC |
| 76 | lh_postcentral_part5 | Cortical | -2.984 | 3.698E-03 | 1.001E-02 | HE < HC |
| 322 | Right-Thalamus | Subcortical | -2.985 | 3.690E-03 | 1.001E-02 | HE < HC |
| 173 | rh_inferiorparietal_part5 | Cortical | -2.964 | 3.925E-03 | 1.053E-02 | HE < HC |
| 100 | lh_rostralmiddlefrontal_part2 | Cortical | -2.959 | 3.983E-03 | 1.060E-02 | HE < HC |
| 75 | lh_postcentral_part4 | Cortical | -2.950 | 4.093E-03 | 1.080E-02 | HE < HC |
| 298 | rh_supramarginal_part4 | Cortical | -2.930 | 4.343E-03 | 1.137E-02 | HE < HC |
| 212 | rh_middletemporal_part5 | Cortical | -2.926 | 4.389E-03 | 1.140E-02 | HE < HC |
| 127 | lh_superiorparietal_part6 | Cortical | -2.923 | 4.435E-03 | 1.143E-02 | HE < HC |
| 254 | rh_precuneus_part7 | Cortical | -2.898 | 4.758E-03 | 1.216E-02 | HE < HC |
| 177 | rh_inferiorparietal_part9 | Cortical | -2.878 | 5.043E-03 | 1.279E-02 | HE < HC |
| 270 | rh_superiorfrontal_part5 | Cortical | -2.818 | 5.998E-03 | 1.509E-02 | HE < HC |
| 78 | lh_postcentral_part7 | Cortical | -2.815 | 6.046E-03 | 1.509E-02 | HE < HC |
| 90 | lh_precentral_part9 | Cortical | -2.810 | 6.126E-03 | 1.517E-02 | HE < HC |
| 252 | rh_precuneus_part5 | Cortical | -2.804 | 6.244E-03 | 1.535E-02 | HE < HC |
| 149 | lh_insula_part1 | Cortical | -2.800 | 6.301E-03 | 1.537E-02 | HE < HC |
| 269 | rh_superiorfrontal_part4 | Cortical | 2.755 | 7.161E-03 | 1.734E-02 | HE > HC |
| 286 | rh_superiorparietal_part8 | Cortical | -2.750 | 7.258E-03 | 1.744E-02 | HE < HC |
| 1 | lh_bankssts_part1 | Cortical | -2.724 | 7.803E-03 | 1.861E-02 | HE < HC |
| 196 | rh_lateralorbitofrontal_part2 | Cortical | 2.719 | 7.925E-03 | 1.876E-02 | HE > HC |
| 205 | rh_medialorbitofrontal_part1 | Cortical | 2.705 | 8.230E-03 | 1.920E-02 | HE > HC |
| 299 | rh_supramarginal_part5 | Cortical | -2.705 | 8.222E-03 | 1.920E-02 | HE < HC |
| 136 | lh_superiortemporal_part5 | Cortical | -2.698 | 8.388E-03 | 1.929E-02 | HE < HC |
| 214 | rh_parahippocampal_part1 | Cortical | -2.698 | 8.387E-03 | 1.929E-02 | HE < HC |
| 216 | rh_paracentral_part1 | Cortical | -2.674 | 8.964E-03 | 2.047E-02 | HE < HC |
| 268 | rh_superiorfrontal_part3 | Cortical | -2.666 | 9.175E-03 | 2.081E-02 | HE < HC |
| 180 | rh_inferiortemporal_part2 | Cortical | -2.637 | 9.930E-03 | 2.236E-02 | HE < HC |
| 41 | lh_lateralorbitofrontal_part1 | Cortical | -2.582 | 1.152E-02 | 2.575E-02 | HE < HC |
| 295 | rh_supramarginal_part1 | Cortical | -2.523 | 1.346E-02 | 2.989E-02 | HE < HC |
| 278 | rh_superiorfrontal_part13 | Cortical | -2.520 | 1.357E-02 | 2.993E-02 | HE < HC |
| 250 | rh_precuneus_part3 | Cortical | -2.482 | 1.502E-02 | 3.289E-02 | HE < HC |
| 54 | lh_middletemporal_part1 | Cortical | -2.469 | 1.554E-02 | 3.381E-02 | HE < HC |
| 139 | lh_supramarginal_part1 | Cortical | -2.449 | 1.636E-02 | 3.524E-02 | HE < HC |
| 217 | rh_paracentral_part2 | Cortical | -2.448 | 1.641E-02 | 3.524E-02 | HE < HC |
| 51 | lh_medialorbitofrontal_part1 | Cortical | 2.428 | 1.728E-02 | 3.685E-02 | HE > HC |
| 174 | rh_inferiorparietal_part6 | Cortical | -2.419 | 1.769E-02 | 3.747E-02 | HE < HC |
| 225 | rh_parstriangularis_part3 | Cortical | -2.413 | 1.795E-02 | 3.777E-02 | HE < HC |
| 48 | lh_lingual_part4 | Cortical | 2.400 | 1.856E-02 | 3.856E-02 | HE > HC |
| 312 | Left-Hippocampus | Subcortical | -2.400 | 1.854E-02 | 3.856E-02 | HE < HC |
| 246 | rh_precentral_part8 | Cortical | -2.392 | 1.896E-02 | 3.913E-02 | HE < HC |
| 104 | lh_rostralmiddlefrontal_part6 | Cortical | -2.381 | 1.945E-02 | 3.986E-02 | HE < HC |
| 202 | rh_lingual_part4 | Cortical | 2.379 | 1.956E-02 | 3.986E-02 | HE > HC |
| 95 | lh_precuneus_part5 | Cortical | -2.374 | 1.981E-02 | 4.012E-02 | HE < HC |
| 16 | lh_inferiorparietal_part1 | Cortical | -2.368 | 2.011E-02 | 4.047E-02 | HE < HC |
| 240 | rh_precentral_part2 | Cortical | -2.366 | 2.025E-02 | 4.049E-02 | HE < HC |
| 194 | rh_lateraloccipital_part9 | Cortical | -2.358 | 2.063E-02 | 4.100E-02 | HE < HC |
| 129 | lh_superiorparietal_part8 | Cortical | -2.355 | 2.081E-02 | 4.110E-02 | HE < HC |
| 84 | lh_precentral_part3 | Cortical | -2.343 | 2.141E-02 | 4.204E-02 | HE < HC |
| 93 | lh_precuneus_part3 | Cortical | -2.326 | 2.239E-02 | 4.370E-02 | HE < HC |
| 186 | rh_lateraloccipital_part1 | Cortical | -2.323 | 2.253E-02 | 4.370E-02 | HE < HC |
| 226 | rh_pericalcarine_part1 | Cortical | 2.294 | 2.426E-02 | 4.677E-02 | HE > HC |

**Supplementary Table 2. Functional disease epicenter ranking in HE**

| **Rank** | **Region ID** | **Region Name** | **Region Type** | **Correlation Coefficient** | **P_spin_** |
| --- | --- | --- | --- | --- | --- |
| 1 | 68 | lh_parstriangularis_part1 | Cortical | -0.2999 | 0.0082 |
| 2 | 66 | lh_parsopercularis_part3 | Cortical | -0.2800 | 0.0094 |
| 3 | 44 | lh_lateralorbitofrontal_part4 | Cortical | -0.2755 | 0.0077 |
| 4 | 65 | lh_parsopercularis_part2 | Cortical | -0.2507 | 0.0099 |
| 5 | 314 | Left-Putamen | Subcortical | -0.2254 | 0.0383 |
| 6 | 143 | lh_supramarginal_part5 | Cortical | -0.2215 | 0.0368 |
| 7 | 116 | lh_superiorfrontal_part8 | Cortical | -0.2156 | 0.0393 |
| 8 | 64 | lh_parsopercularis_part1 | Cortical | -0.2085 | 0.0476 |

**Supplementary Table 3. Structural disease epicenter ranking in HE**

| **Rank** | **Region ID** | **Region Name** | **Region Type** | **Correlation Coefficient** | **P_spin_** |
| --- | --- | --- | --- | --- | --- |
| 1 | 112 | lh_superiorfrontal_part4 | Cortical | -0.3013 | 0.0054 |
| 2 | 270 | rh_superiorfrontal_part5 | Cortical | -0.2981 | 0.0026 |
| 3 | 66 | lh_parsopercularis_part3 | Cortical | -0.2906 | 0.0042 |
| 4 | 120 | lh_superiorfrontal_part12 | Cortical | -0.2787 | 0.0145 |
| 5 | 110 | lh_superiorfrontal_part2 | Cortical | -0.2707 | 0.0030 |
| 6 | 85 | lh_precentral_part4 | Cortical | -0.2697 | 0.0205 |
| 7 | 64 | lh_parsopercularis_part1 | Cortical | -0.2668 | 0.0054 |
| 8 | 65 | lh_parsopercularis_part2 | Cortical | -0.2666 | 0.0051 |
| 9 | 81 | lh_posteriorcingulate_part2 | Cortical | -0.2631 | 0.0317 |
| 10 | 151 | lh_insula_part3 | Cortical | -0.2623 | 0.0273 |

****Supplementary Table 4. Quantitative comparison between DK308 main analyses and Schaefer400 sensitivity analyses****

| **Analysis** | **DK308 main analysis** | **Schaefer400 sensitivity analysis** | **Interpretation** |
| --- | --- | --- | --- |
| FDR-significant regions | 167/322 regions | 193/414 regions | Both parcellations showed widespread gray matter abnormalities. |
| Direction of significant effects | 154 lower and 13 higher in HE | 181 lower and 12 higher in HE | Both parcellations showed predominantly lower cortical thickness or subcortical volume in HE. |
| Cortical FC hub association | r = 0.14, P_spin_ = 0.343 | r = 0.07, P_spin_ = 0.650 | No significant cortical functional hub-preferential pattern in either parcellation. |
| Cortical SC hub association | r = 0.08, P_spin_ = 0.457 | r = −0.06, P_spin_ = 0.576 | No significant cortical structural hub-preferential pattern in either parcellation. |
| Subcortico-cortical FC hub association | r = −0.12, P_shuf_ = 0.697 | r = −0.14, P_shuf_ = 0.633 | No significant subcortico-cortical functional hub association in either parcellation. |
| Subcortico-cortical SC hub association | r = −0.23, P_shuf_ = 0.451 | r = −0.20, P_shuf_ = 0.506 | No significant subcortico-cortical structural hub association in either parcellation. |
| Cortical FC network-neighborhood effect | r = −0.07, P_spin_ = 0.643 | r = −0.08, P_spin_ = 0.614 | Functional neighborhood effect remained nonsignificant across parcellations. |
| Cortical SC network-neighborhood effect | r = 0.58, P_spin_ = 0.004 | r = 0.62, P_spin_ = 0.040 | Structural neighborhood effect was reproduced across parcellations. |
| Subcortical FC network-neighborhood effect | r = 0.00, P_shuf_ = 1.000 | r = −0.13, P_shuf_ = 0.663 | No significant subcortical functional neighborhood effect in either parcellation. |
| Subcortical SC network-neighborhood effect | r = −0.09, P_shuf_ = 0.746 | r = −0.06, P_shuf_ = 0.842 | No significant subcortical structural neighborhood effect in either parcellation. |

Abbreviations: FDR = false discovery rate; FC = functional connectivity; SC = structural connectivity.

References

1. Alexander-Bloch AF, Shou H, Liu S, Satterthwaite TD, Glahn DC, Shinohara RT, et al. On testing for spatial correspondence between maps of human brain structure and function. Neuroimage. 2018;178:540-51.

2. Larivière S, Rodríguez-Cruces R, Royer J, Caligiuri ME, Gambardella A, Concha L, et al. Network-based atrophy modeling in the common epilepsies: A worldwide ENIGMA study. Sci Adv. 2020;6(47):eabc6457.

3. Glasser MF, Sotiropoulos SN, Wilson JA, Coalson TS, Fischl B, Andersson JL, et al. The minimal preprocessing pipelines for the Human Connectome Project. Neuroimage. 2013;80:105-24.

4. Tournier JD, Smith R, Raffelt D, Tabbara R, Dhollander T, Pietsch M, et al. MRtrix3: A fast, flexible and open software framework for medical image processing and visualisation. Neuroimage. 2019;202:116137.

5. Betzel RF, Griffa A, Hagmann P, Mišić B. Distance-dependent consensus thresholds for generating group-representative structural brain networks. Netw Neurosci. 2019;3(2):475-96.
